# Edible Insect Production in Thailand: Sustainable Supply Chain Management

**DOI:** 10.3390/insects16080827

**Published:** 2025-08-08

**Authors:** Sasiprapa Krongdang, Karthikeyan Venkatachalam, Thararat Chitov, Sutee Wangtueai, Jittimon Wongsa, Thanya Parametthanuwat, Narin Charoenphun

**Affiliations:** 1 Faculty of Science and Social Sciences, Burapha University Sa Kaeo Campus, Sa Kaeo 27160, Thailand; sasiprapa.kr@buu.ac.th; 2 Faculty of Innovative Agriculture, Fisheries and Food, Prince of Songkla University, Surat Thani Campus, Makham Tia, Mueang, Surat Thani 84000, Thailand; karthikeyan.v@psu.ac.th; 3 Department of Biology, Faculty of Science, Chiang Mai University, Chiang Mai 50200, Thailand; thararat.chitov@cmu.ac.th; 4 Environmental Science Research Center (ESRC), Faculty of Science, Chiang Mai University, Chiang Mai 50200, Thailand; 5 School of Agro-Industry, Faculty of Agro-Industry, Chiang Mai University, Mae Hia, Muang, Chiang Mai 50100, Thailand; sutee.w@cmu.ac.th; 6 Faculty of Industrial Technology and Management, King Mongkut’s University of Technology North Bangkok (Prachinburi Campus), Muang 25230, Thailand; jittimon.w@itm.kmutnb.ac.th (J.W.); thanya.p@itm.kmutnb.ac.th (T.P.); 7 Food and Agro-Industry Research Center, King Mongkut’s University of Technology North Bangkok, Bangkok 10800, Thailand; 8 KMUTNB Techno Park Prachinburi, King Mongkut’s University of Technology North Bangkok (Prachinburi Campus), Muang 25230, Thailand; 9 Faculty of Science and Arts, Burapha University Chanthaburi Campus, Chanthaburi 22170, Thailand

**Keywords:** supply chain, edible insects, sustainability, BCG model, TOWS matrix, policy

## Abstract

This study aims to foster a guideline for sustainable supply chain management for edible insect production through a comprehensive review and critical analysis of the current situation, focusing especially on Thailand’s perspective. It addresses the traditional culture and modern practice of insect consumption and how these factors affect current production for commercial purposes. The advantages of nutritional values and farming with edible insects over traditional livestock farming are highlighted. A threats, opportunities, weaknesses, and strengths (TOWS) matrix analysis, producing action-oriented strategies, was based on an analysis of the strengths, weaknesses, opportunities, and threats (SWOT) of Thailand’s edible insect industry. The strategies include enhancement of production and product quality, innovation of intelligent production techniques and agriculture, fortification of branding and product development, encouragement of research and development investment, and the promotion of local product identity. The analysis emphasizes the value of sustainable supply chain management and advocates for the advancement of the bio-circular green (BCG) economy strategy for the edible insect industry. It also emphasizes the importance of stakeholder collaboration and implementing regulations to ensure food safety and sustainable production.

## Simple Summary

The edible insect sector in Thailand has been steadily growing in economic value; however, gaps remain in knowledge regarding its sustainability and supply chain management. This study explores opportunities for the sustainable supply chain management of edible insects by applying a TOWS matrix analysis derived from a SWOT assessment of Thailand’s edible insect industry. The analysis identifies actionable strategies to help insect farm businesses enhance product quality, encourage innovation, and seize opportunities in global markets. Furthermore, the development of the insect sector also aligns with Thailand’s bio-circular green (BCG) economy model and contributes to the Sustainable Development Goals (SDGs), highlighting insects as innovative, sustainable alternative protein sources to traditional livestock farming.

## 1. Introduction

**Figure 1:**
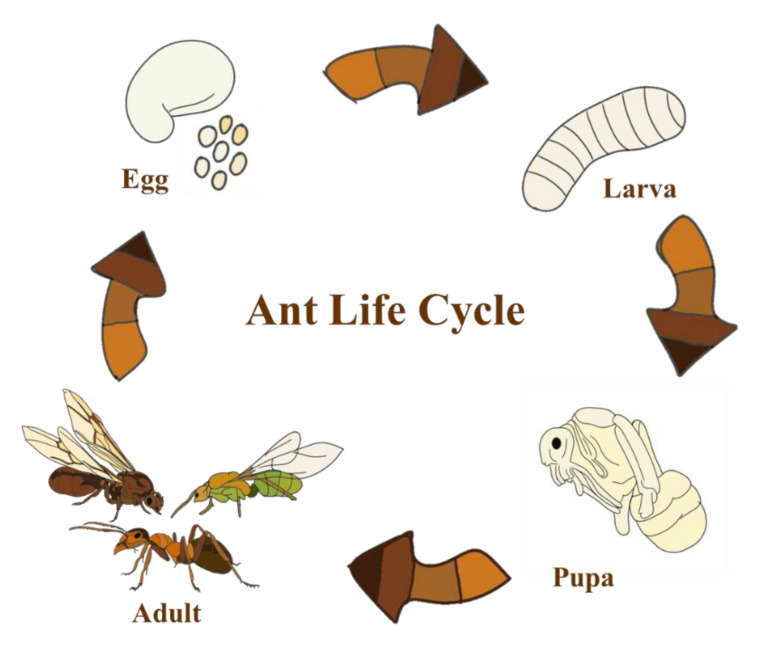
Life cycle of ants.

Insects are the most abundant group of animals worldwide. They are commonly found in natural habitats and have traditionally made up a part of the diet for many cultures. Insects in many stages of their life cycle, including eggs, larvae, pupae, and adults, can be used as food. For example, with ants (Figure 1), larvae and pupae of weaver ants are popularly consumed in Southeast Asia, particularly Thailand and Laos (known in Thai as Kai Mod Daeng) [1]. Adult ants, such as winged leaf-cutter ants and weaver ants, are widely consumed in South American countries [2].

The recognition of insect species that are edible is geographically specific and has been passed on through generations. In many parts of the world, particularly Asia, Africa, Oceania, and Latin America, insect consumption is an integral aspect of traditional culture and reflects longstanding local knowledge [3,4,5]. In some other regions, such as North America, although insect consumption has been historically practiced among North American natives and some colonists, there is a renewed interest in the consumption of edible insects in modern societies [6,7]. The introduction or reintroduction of insects consuming as food has led to the establishment of control by the countries’ food authorities, which take different control measures for assuring its safety. For example, the production of insects for human consumption in the United States is allowed by the United States Food and Drug Administration (USA FDA), provided it meets general food safety standards [7,8]; while in Singapore, the Singapore Food Agency (SFA) provided guidelines for the production of insects as food and a list of insect species (with their developmental stages) approved for human consumption [9]. In the EU, the European Food Safety Authority (EFSA) is the authority that takes responsibility for controlling food safety standards [8,10,11]. Recently, the microbiological quality and bacterial communities of frozen edible insects from Thailand have been analyzed to address safety concerns, characterize their native flora, and establish criteria for foodborne-pathogen monitoring in order to comply with the EU’s export regulations [12]. The increasing popularity of edible insects can be attributed to their high nutritional value. Their protein content is comparable to that of pork, fish, chicken, and beef [3,6]. Many edible insects contain high levels of fiber, vitamin B2, niacin, and beneficial fatty acids, making them an appropriate substitute food source [13,14,15]. Livestock production and its large emissions of greenhouse gases, particularly methane, which has a greater global warming potential than carbon dioxide, clearly have environmental impacts. Reducing reliance on animal sources of protein and energy is essential, as livestock production contributes over 14.5 percent of greenhouse gas emissions [16]. Therefore, raising edible insects is an interesting alternative to help reduce greenhouse gas emissions from livestock, which will help mitigate the impact of climate change, reduce the risk of natural disasters, and also have positive effects on human health and the environment.

Edible insect farming, in contrast to traditional livestock farming, produces lower greenhouse gas emissions, requires less land and water, has a reduced life cycle, and shows a better feed conversion rate [17,18]. The United Nations has acknowledged the necessity of mitigating greenhouse gas emissions and has planned Sustainable Development Goals (SDGs), specifically the 13th goal, which emphasizes the urgency of taking action against climate change and its effects [19]. The promotion of insect farming, therefore, is a way of addressing the need for sustainable food production [18]. Moreover, edible insects serve as an eco-friendly and nutritious protein source, playing a significant role in reducing food insecurity and hunger, thereby directly supporting the SDG 2 (Zero Hunger) [18,19].

Edible insects can be processed into different products, such as ready-to-eat whole insects, frozen insects, and dried powdered insects, which are used as food ingredients [20]. Since many consumers are not accustomed to the whole-insect appearance, the development of food products with modified forms of edible insects can reduce apprehension towards whole-insect appearance and enhance consumer acceptance of edible insect products, thus increasing the market sizes. Additional areas of emphasis on product development involve maintaining their nutritional values and ensuring their quality and safety throughout the food supply chain [21]. These can be achieved through careful design of food processing methods, which may include the application of advanced processing technologies [22]. In Thailand, examples of edible insects traditionally consumed comprise bamboo borers, house crickets, mole crickets, locusts, silkworms (silk moth pupae), palm weevil larvae, and red ant (weaver ant) larvae and pupae [23]. Insect consumption is growing among the Thai population, and there has been an increasing demand for export recently. The growing popularity of insect consumption has notable commercial implications, leading to a transition from wild insect harvesting to farming and industrial production, with certain products directed toward export markets [24]. This new era of large-scale production of edible insects and the expanding export markets calls for specific guidance throughout the production steps in the food supply chain and the establishment of food criteria that aim to control the quality and safety of edible insect products [25,26]. With the variety of edible insect types, it is important to develop strategies and recommend practices that take specific insect species, as well as farming and processing methods, into consideration. In cricket production in Thailand, Good Agricultural Practices (GAPs) for cricket farms were established eight years ago. The most popular cricket species raised by farmers is the field cricket (*Gryllus bimaculatus* De Geer). It is common to build gable-fronted houses, use ponds made from smartboards, and raise an average of 17.08 cricket boxes per owner. One generation of cricket farming takes 30–40 days. Most cricket farms use ready-made cricket feed. The most common type of cricket farming is for fresh crickets, which are sold for consumption within the community and to wholesalers. Cricket farming can be performed six–seven times per year. In winter, crickets eat less food, which reduces their growth rate [27]. The review aims to develop sustainable management strategies and offer recommendations for the supply chain in Thailand by analyzing current conditions, including both its advantages and challenges.

## 2. SWOT–TOWS Analysis for Development of Edible Insect Business Strategy in Thailand

A SWOT analysis was conducted to evaluate the environment and potential of edible insect business in Thailand. It explores internal factors, including strengths and weaknesses, and external factors, including opportunities, difficulties, restrictions, and operational hazards and potential threats or significant challenges [28].

The analysis from the two viewpoints would provide a comprehensive understanding of the environment and the business itself and identify opportunities and obstacles. Examining a range of external and internal factors would help gain insight into the changes that have taken place outside, as well as the trends that will affect these /changes in the future [29]. The effects of these changes on the business and its capabilities to respond to these changes would also be considered. This information will help develop and implement best practices for producing edible insects in Thailand. The results of the SWOT analysis are shown in Table 1.

The analysis of the environment and potential for edible insect business in Thailand leads to the proposal of strategy through a matching process using the TOWS Matrix. This approach is based on two primary patterns: matching and converting, which involve aligning internal factors (weaknesses and strengths) with external factors (obstacles and opportunities) to identify the most appropriate strategies-classified into four distinct strategies. Table 2 illustrates that matching involves aligning identical characteristics. (1) SO strategy: Strengths and Opportunities (Maxi–Maxi), which refers to “opportunities and strengths,” and (2) WT strategy: Weaknesses and Threats (Mini–Mini), which pertain to “obstacles and weaknesses.” The Converting segment involves a correlation of various features, including the WO strategy, which addresses weaknesses and opportunities (Mini–Maxi), and the ST strategy, which focuses on strengths and threats (Maxi–Mini) [44].

## 3. Managing the Supply Chain of Edible Insects for Sustainability

Environmental, social, and governance (ESG) criteria are a concept related to sustainable corporate development, which is an abbreviation of environmental, social, and governance. ESG is currently popular among investors around the world because it is a concept that investors use to consider investing. It focuses on doing business that takes into account three main responsibilities: environment, society, and governance. Environment is a criterion that takes into account the responsibility of the company towards the environment. Social is a criterion used to measure how the company manages relationships and communicates with employees, suppliers, customers, or stakeholders. Governance is a principle used to measure how the company manages relationships in terms of governance for effective, transparent, auditable management and taking into account stakeholders. The ESG concept helps build credibility for businesses by reflecting the role of responsibility of the business towards stakeholders and presenting the performance in developing the business to grow sustainably [51]. A sustainable supply chain incorporates ethics and environmental, social responsibility into its operations, keeping accountability and awareness of environmental, social, and governance affects from upstream to downstream [18,52]. The fundamental components of a sustainable insect manufacturing supply chain are as follows:

(1) Green supply chain

A green supply chain integrates environmental responsibility to supply chain management across production, processing, and distribution stages (Table 3). To minimize waste and enhance efficiency, it is essential to evaluate approaches that will benefit the environment, stakeholders, or operators in the insect industry to minimize the potential risks associated with each activity [25].

(2) Transparent supply chain

A transparent supply chain signifies that an insect business operator holds the capacity and readiness to reveal information regarding the origins, labor conditions, and related practices throughout the supply chain, from production to distribution. In doing this, the business operator has to have a clear and thorough awareness of activities occurring at each phase of its supply chain and a commitment to communicate this information to internal and external stakeholders, including customers, investors, and regulators [25,32,38].

(3) Circular supply chain

**Figure 2:**
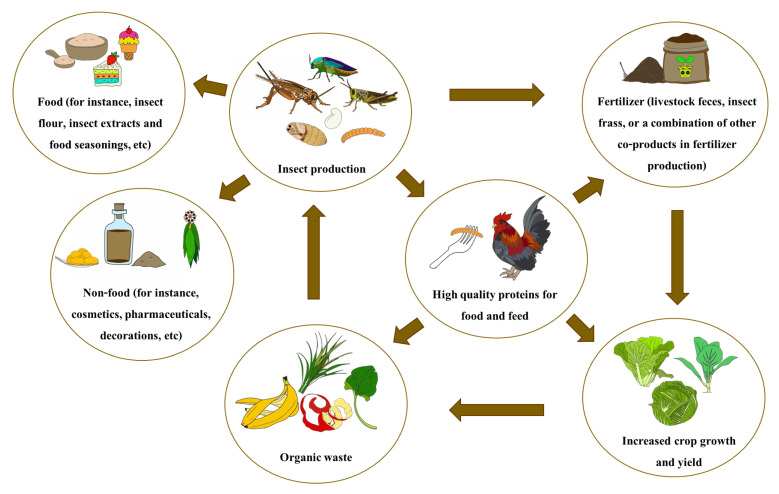
Circular supply chain of edible insects.

In a circular supply chain, organic materials are converted into commercially valuable products. This material recycling may reduce production costs (Figure 2), aiming to enhance supply efficiency [3,53]. A circular supply chain also minimizes the adverse environmental effects of insect farming. Such practices will yield economic advantages for producers by enhancing sales, as consumers are increasingly mindful of the environmental implications of food production. To effectively manage supply chains, all stakeholders must be committed to sustainability [25].

## 4. Pursuing the Bio-Circular Green (BCG) Economy Approach

**Figure 3:**
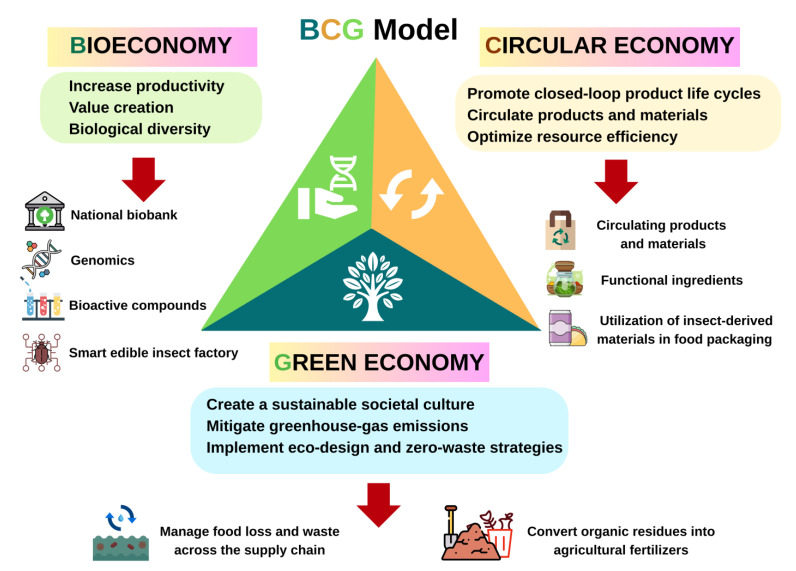
Establishing sustainability for edible insect businesses in accordance with the BCG model.

The bio-circular green economy (BCG economy) represents an SDG economic model [54]. Alongside aiding communities and farmers in enhancing productivity, the principles of science, technology, and innovation also support entrepreneurs in developing innovative or high-value products and services that enhance long-term competitiveness. BCG promotes innovation within the sustainable economy by emphasizing waste management in production and consumption, facilitating the recycling of raw materials throughout these processes, and advancing upcycling in production [55]. These advancements include eco-design and zero waste, which aim to eliminate waste through the design of products and production processes, as well as promote reuse, refurbishment, and sharing [56]. Establishing sustainability for edible insect businesses under the BCG model is shown in Figure 3.

## 5. Components of BCG Economy

### 5.1. Bioeconomy

Bioeconomy is the use of biological resources, whether plant, animal, or other organisms, as well as products, in the discovery of important new substances, but systematically, such as the creation of a national biobank of edible insects using modern knowledge. Numerous animal species have been cataloged for conservation, and their genetic resources are being leveraged through reproductive biotechnologies, biobanks, and conservation breeding programs (RBCs) to advance sustainability goals [57]. The Bio-Resource Bank supports the conservation and sustainable use of insects. Bio-resource information research and development is used to maximize the benefits of information. The role of the Data Bank is to promote the useful use of valuable information on national bio-resources [58]. The recognition of genetic materials or genomics, the management of the synthesis and identification of important active ingredients or functional ingredients, the boosting of product volume with Smart Edible Insect Factory (SEIF) technology, which manages all environmental parameters to achieve a higher yield, and the utilization of a fast, high-throughput screening process are some advantages of this technology [33,59,60]. The SEIF technology could integrate the Internet of Things (IoT)-enabled sensors and automated controls to continuously monitor and adjust temperature, humidity, and ventilation; high throughput phenotyping and genotyping platforms [61] rapidly assess growth rates, biomass composition, and health metrics across large cohorts; advanced data analytics [60] and AI-driven predictive models analyze these data to fine tune protocols and forecast yield performance [62]; and blockchain-based traceability systems record every step from substrate sourcing through harvest, thereby ensuring transparency, compliance with food safety standards, and swift product recall when necessary [32]. In addition to the technology that enhances, changes, or boosts the efficacy of the obtained material, functional components also come with standardized testing and control over each step of the production process [5]. Bioeconomy focuses on the useful use of bio-resources with the maintenance of environmental balance by supporting technological advances in a wide range of fields, enhancing efficiency, and promoting innovation. Therefore, bioeconomy places greater emphasis on the commercial exploitation of biotechnology to reduce long-term economic costs, which is regarded as a new-current economy driven by innovation, technology, and creativity, with social and environmental sustainability in mind [63,64,65]. Guidelines for driving the bioeconomy of edible insects: Bioeconomy refers to the systematic use of biological resources, including plants, animals, and other organisms, together with their derivatives, to produce valuable new substances [66]. It emphasizes the effective utilization of biological resources through technology, creativity, and innovation. It prioritizes mitigating long-term economic expenses while considering social and environmental sustainability [63,66].

In edible insects, creating a bio-resource bank from the diverse array of edible insects found in tropical regions exemplifies an application of the bioeconomy. This initiative will facilitate both the preservation and sustainable utilization of these insects while also advancing research and development based on bio-resource information [57]. The effective application of bio-resources, paired with efficient data utilization, can drive progress in both academic and industrial sectors. For instance, it can enable the identification of genetic materials or genomics [33,61]. It can lead to the discovery and synthesis of valuable active compounds or functional ingredients [59]. It can enhance product volume through SEIF technology [60] that regulates all environmental parameters to increase yield and implement a rapid, high-throughput screening process. The application of technology that improves, modifies, or amplifies the usefulness of obtained materials and functional components is also supported by standardized testing and monitoring throughout each phase of the production process [5]. The following are guidelines for promoting the bioeconomy of edible insects:

(1) Apply innovation to advance the country’s food industry with technology and innovation management of production processes, products, and services. Such goals can be achieved through transferring knowledge and technology regarding edible insects from research to industry, and the economic impact can be increased by commercial production [67].
(2) Utilize information technology to gather primary data from edible insect farmers (local and nationwide) regarding edible insect species, production capacity, costs, disease information, and external variables such as temperature, humidity, and feed. This data is essential for establishing strategy and policy and promoting entrepreneurs, which includes both the facilitation and elimination of barriers, as well as transferring advanced technology and skills to entrepreneurs in the edible insect sector [47,68].
(3) Facilitate cultivation of edible insects that align with local potential and market demands for integration of technology and innovation. Utilization of local resources and regional biodiversity can minimize the costs associated with the transportation of production elements. This can increase the competitiveness of local businesses [3].
(4) Establish edible insect breeds characterized by high nutritional value, substantial yield, genetic uniformity, disease resistance, and endurance at environmental fluctuations. Technological advancements can facilitate these processes. The quantity and quality of insect harvests depend upon the selected species or breeds appropriate for commercial production [69].
(5) Encourage edible insect farming that is aligned with market demands. Entrepreneurs have to evaluate consumer marketing data, both nationally and internationally, prior to investing in production, to guarantee commercial viability [49,70].

### 5.2. Circular Economy

A renewable economy requires the most valuable use of resources in every process. Nearly every step should aim to eliminate waste entirely or maintain it at a minimal level [71]. To maximize the efficiency of all processes, a renewable economy is a planned economy that allows all resources in the production system to be restored and reused to address future resource shortages. A sustainable economy system is an industrial system that restores or revives various materials in the product life cycle instead of being dumped at the end of consumption [72]. Renewables lead to the materials that make up these products to create new value. A renewed cycle is continuous in that it does not produce waste. It also focuses on preserving and balancing natural resources, along with reducing external efficiencies and designs [73].

The extraction of essential elements from edible insects and the use of insect-derived materials in food packaging are two instances of manufacturing methods that have undergone a circular economy [74]. Insects are expected to be the major source of protein in the future because they can be consumed as food [75]. Besides their nutritional value, edible insects contain various helpful compounds, such as proteins, amino acids, minerals, vitamins, and easily absorbed fatty acids, which can offer significant advantages to humans [22,75]. Insects consume various intriguing active substances, such as steroid materials, lecithin, cordycepin (sex-attractant hormones), polysaccharides, interferon, chitin, chitosan, and antimicrobial peptides [6,14,15]. The biological advantages of these vital compounds include immunosuppression, tumor inhibition, control over intestinal function, decrease in weariness, anti-oxidation, prevention of flu, improvement of sleep, growth and development, and lowered blood pressure and sugar levels [59,76]. Bioactive compounds from edible insects have been used as sources of novel medications in healthcare, agriculture, food technology, pollution remediation, and textile industries, besides serving as animal feed [15,18,20,23,38]. To ensure the sustainability of the insect industry, additional expansion is necessary to explore and use essential compounds in edible insects. The edible insect industry has benefited from sustainable replenishment opportunities, robust government policy support, funding, substantial consumer demand, favorable social dynamics, and advocacy from organizations [59].

The recycling of products and materials derived from insects and their reuse in food packaging manufacturing is an interesting choice. Insects can produce biological substances such as chitin, keratin, and insect proteins with physical, chemical, and biological properties suitable for use as food packaging materials. These materials exhibit outstanding strength, elasticity, waterproofness, and degradation capabilities. Insect materials may also have antimicrobial effects, which can increase the shelf life of food [77]. The production of biodegradable plastic films containing insect ingredients adds value to the use of proteins produced by waste-depleting insects. This process contributes to a renewable economy. Examples of cyanide extraction from sunflowers to produce bioplastics not only increase the value of food but also help replace food objects and bacteria. The outcome is genuine quality packaging that is still needed to ensure that these products are developed in an international environment [78].

The fundamental principle of a circular economy is an understanding of how nature was created and operates. Nature, with its complete recycling mechanism for energy, minerals, and materials, is the most efficient circular production system and wastes nothing. All matter, as they say, will always exist in this world. By boosting the percentage of recycling and lowering waste, the circular economy offers the following benefits: it stabilizes resource use for maximum benefit and efficiency; it also fosters innovation, opens up new business opportunities, boosts employment, and lessens environmental impact in additional ways [20].

(1) Promote the creation of added value from edible insect processing by developing functional compounds using extraction technology and isolating key substances from insect raw materials, along with nutritional value analysis and safety assessment of such essential substances to obtain quality functional substances, such as proteins, fats, and chitin [22,75].
(2) Promoting the use of edible insect materials as ingredients in traditional food production and developing new food products with sensory qualities that are acceptable to consumers in terms of appearance, texture, smell, delicious taste, and complete nutritional value to obtain alternative insect protein products that are in harmony with consumption methods (alignment) and are alternative protein foods to create food security for the world in the future [79,80].
(3) Encouraging consumer business entrepreneurs to provide export knowledge to push policies to expand trade opportunities and exports abroad, as well as supporting the state to build a partner database for conducting export business to ensure competitiveness, stability, and sustainability [81,82].
(4) Developing a critical and necessary infrastructure to upgrade the country’s insect food industry so that food enterprises can compete in the global market, especially medium-sized or small enterprises that still lack investment funding. The state should also create systematic opportunities for accessing funding to promote the infrastructure and facilitate the integration of SME groups, large-scale agriculture, or associations, which will increase negotiating power and production capacity [83,84].
(5) To ensure the timely progress of the food industry both today and in the future, we must develop and improve various regulations related to food derived from edible insects. A balance must be established between setting new standards to protect consumer safety and economic development in the country’s food industry [8].
(6) Promoting legal and regulatory knowledge of food safety and standards for business operators on edible insects [8].
(7) Promote and push knowledge of product standards and edible insect farm standards to build trust in products and processed products in a complete cycle, from upstream, midstream, and downstream, especially to establish trust in trade brands at the provincial, regional, and global levels [23]. According to the latest information, the ACFS has announced three new GAP standards for Thailand, covering dried crickets, frozen crickets, and BSF farming [43], which should raise awareness among of all relevant stakeholders and concretely upgrade export standards.
(8) Supported Food Safety Assessment. Processed edible insect food products have not yet been standardized or regulated. It is necessary to promote pre-production and commercial safety data collection and assessments. Insects that may have been processed using technology under different temperatures and environmental conditions will present distinct qualities or standard risks. This affects the nutritional value and safety of the product. Some insects contain allergens, microorganisms, and toxins that may affect consumers of insect-processed foods [85].

### 5.3. Green Economy

The green economy is a development approach that emphasizes balanced economic growth. Stability and sustainability are simultaneously achieved through social and environmental protection. Green economics is a concept of economic development that aims to solve the imbalances caused by the growing global population, which has increased the need for housing, agricultural land, energy, and food. There is a significant decrease in resources when economic expansion is achieved by depleting the existing resources. Waste deposited into the ecosystem at this rate is unsustainable [20]. Edible insect production in farm systems can reduce greenhouse gases in the agricultural sector more than livestock farming. With the increasing world population, consumers’ demand, and limited agricultural space, there is an urgent need to find alternatives to ordinary meat products. Livestock production is a major cause of anthropogenic climate change. As insects require less food for growth, they reduce greenhouse gas emissions in the feed production process [16,17,18,19]. Reducing meat consumption and using alternative protein sources can promote sustainability. Insects have been promoted as high-protein food sources for humans and animals globally [86]. Therefore, sustainable insect-harvesting practices must be developed and implemented. Edible insect varieties can be preserved and promoted through forest management. Insect rearing can be performed on small or large-scale industrial farms and is more environmentally friendly than livestock production [16,17,18,19]. The important environmental advantages of insect farming compared with animal production are the following: (1) less land and water use; (2) less melting gas emissions; (3) insects are highly efficient in food processing; (4) insects can convert organic fertilizers, low value, into high-quality food or animal feed. For example, after BSF grows by eating organic materials or organic fertilizers, the larvae accumulate proteins and fats in their body. The proteins in insects are highly nutritious, suitable for use as a food source for pets or even as food for humans in some cases [87]; and (5) some insects may be used as animal or aquatic feed. For example, they can replace strawberries, which are scarce and more expensive; however, they are increasingly more expensive [88].

Throughout the food supply chain, a decrease in food mass, particularly food for human consumption, is termed food loss. Food loss occurs at the production, harvesting, and processing stages of the food supply chain. Food waste refers to the loss of food occurring at the final stages of the food chain, specifically during retail and consumption. This phenomenon is associated with retail practices and consumer behavior [89]. Uneaten food waste can be used by insects. In addition, the management of food waste, such as through insect biotransformation, can serve as a viable solution for mitigating food waste. This method effectively transforms substantial amounts of food waste into valuable products, such as human food, animal feed, fertilizers, and valuable compounds. About one-third of global food production intended for human consumption, corresponding to approximately 1.3 billion tons annually, results in waste [90]. Numerous insects inherently consume organic waste. Insects can minimize waste by digesting organic materials within their bodies, resulting in the production of valuable biomass for various applications, including food, feed, ingredients, cosmetics, disease treatments, and bioplastics [91,92].

The utilization of insects for waste reduction represents an appealing approach that combines environmentally conscious design with the concepts of a sustainable economy. Food waste serves as a raw material for insect feeding, a process termed biological transformation, which can convert significant amounts of food waste into high-value products, including food, animal fertilizers, and various minerals. Insect farming represents an interesting approach to completing the food value chain. The BSF effectively reduces organic waste. BSF is a popular choice for industrial farming due to its short life cycle. It has an excellent feed conversion rate and can convert and extract nutrients from a variety of organic materials. Insect larvae can reduce food waste by 50–60% and convert it into high-protein biomass. In addition, the nutritional value of BSF larvae is comparable to oilseeds such as hemp, flaxseed, and rapeseed, with a protein content of up to 28% and an oil content of up to 40%. Each of Agriprotein’s facilities currently has the capacity to process 350 tons of food waste per day and produce proteins, oils, and organic soil amendments using BSF. Several other companies and startups around the world are rapidly expanding to solve the food waste problem with insect-based bioprocesses [89]. Edible insect farming has balanced global food sources and local alternatives. This effort will yield a thorough evaluation of the insect industry, extending beyond standard criteria like quality, food safety, and expected return on investment. Alongside the evaluation of economic sustainability, the assessment of environmental and social affects is crucial. Promoting circular and green economies can enhance the sustainability of the food system by repurposing waste from insect farming as fertilizer.

The utilization of insect fat as a feedstock for biodiesel production, along with incorporating insect proteins in human and animal diets, has the potential to decrease livestock imports and lower production costs. The biodiesel production process from BSF generates high-density fats in its body, which can be extracted and used for biodiesel production through the process of transesterification (conversion of fatty acids in oil into esters). After the reaction is complete, the products obtained consist of biodiesel and glycerol, which can be separated using liquid separation techniques such as filtration or precipitation. The separated glycerol can be used for other industrial purposes or in the production of other chemicals as well [93]. Challenges persist, including the selection of suitable breeds, environmental management, and waste management related to feeding requirements and regulations [94]. Incorporating natural pesticides into organic fertilizers may serve as an alternative source of organic fertilizer, decreasing dependence on synthetic chemicals while enhancing agricultural productivity. Cricket frass is now used in the formulation of organic vegetable fertilizers, replacing chemical fertilizers. Research showed that all vegetables grown using organic vegetable fertilizers exhibited similar nutritional values to those containing nitrogen, phosphorus, and potassium (NPK), 15-15-15 compounds [95]. Organic vegetable fertilizers were shown to provide adequate nutrients for the optimal growth of vegetable [95,96].

Promoting the sustainable strengthening of communities through public–private collaboration allows communities to jointly develop community spaces, build careers, and support production or processing edible insects as primary or complementary careers. This initiative aims to increase per capita income while also strengthening minority groups and feeding deprived communities, all with building environmental and safety awareness [97]. Therefore, an economy that develops with consideration for environmental sustainability, appropriate use of resources, awareness of their value, reduction in greenhouse gas emissions, and equal distribution of wealth is one that all countries must use as a guideline for their development.

## 6. Conclusions

In Thailand, the consumption of edible insects is a longstanding practice that offers a sustainable and nutritious alternative to conventional livestock. To facilitate sector expansion, it is essential to address challenges, including customer acceptability and production techniques. To establish an edible insect company, it is essential to implement strategies that include innovation, branding, and research. Managing the supply chain sustainably requires the implementation of ethical and environmentally friendly practices, with a focus on efficiency and waste reduction. The BCG economy model is advocated for promoting sustainability, emphasizing the utilization of biological resources and the adoption of circular economy practices. Food packaging employs materials derived from insects, providing a sustainable protein source rich in beneficial compounds. Intersectoral collaboration is essential for promoting the utilization of insect-based products and achieving sustainable economic, social, and environmental outcomes. The subsequent significant observations that were noted are as follows:

- Edible insects serve as a viable and nutritious alternative to traditional livestock, demonstrating environmental sustainability.
- To expand the market, it is essential to address challenges related to customer acceptability and production techniques.
- The BCG economy model is advocated for promoting sustainability, emphasizing the utilization of biological resources and the adoption of circular economy practices.
- Food packaging uses materials produced from insects, which serve as a sustainable protein supply abundant in helpful chemicals.

The possible strategies for advancing the edible insect industry in Thailand are as follows:

- The application of the BCG economy strategy and SWOT–TOWS analysis are included in the strategies.
- Additionally, emphasis is placed on environmentally responsible and ethical supply chain management, integrating environmental and social responsibility.
- This study shows the effectiveness of circular and green economies in promoting sustainability in the edible insect sector.

## Figures and Tables

**Table 1:** SWOT analysis for edible insect business in Thailand [29].

Strengths (Internal Factors)	Weaknesses (Internal Factors)
**A wide diversity of insect species and types of edible insects available** Thailand has excellent conditions for insect cultivation because of its geographical location and climate. As a result, there is a wide variety of edible insects in Thailand. Owing to the high diversity of insect species and the increasing popularity of insect farming, certain species are available for purchase in all seasons [23]. **Production advantages** The climate in all parts of Thailand supports edible insect farming. Other beneficial environmental aspects include a wide variety of feeds, low farming costs, rapid breeding, and high yields [23]. **Cultural diversity** The consumption of edible insects is part of traditional Thai culture and is unique to each region. Insect consumption and utilization can be a means of passing on indigenous knowledge from one generation to the next. The richness of “biodiversity” together with “cultural diversity” maximizes and improves the value chain of edible insect products [23,30]. **Promotion from government sector** Currently, the government promotes farming and consumption of insects, as they are a rich source of protein and have high economic value. Production of edible insects can be performed at a lower cost than other livestock and is environmentally friendly. They can be manufactured locally and processed into high-quality raw materials, such as protein, fat, and chitin, which are used in food and related industries, such as the pharmaceutical industry [31].	**Small and inconsistent production quantity** A large proportion of edible insect production is still carried out in a traditional manner, meaning that the quantity is small and the quality is inconsistent. Currently, only crickets meet the market demands for edible insects [31]. **Incomplete supply chain** Edible insect production is mostly a family business, small and medium-sized enterprise (SME), or natural harvest business. Because there are no industrial systems in many insect farming areas, there is a gap in the supply chain, which also affects the consistency of primary production [32]. **Control of breeding stocks** There is currently no rational control over the breed because farming is passed down as local knowledge. This process contributes to inbreeding, which raises the possibility of low disease resistance and, over time, a decrease in the size of the insects’ bodies and populations [33]. **Processing technology and marketing** In production at the SME level, there is a limited product variety and a lack of advanced processing methods. A wide variety of customers and expanded markets requires new, creative forms of edible insect products; processing methods that enhance sensory qualities while preserving nutritional values; and more advanced packaging that combines innovation and technology into production, processing, and control [23].
**Opportunities (external factors)**	**Threats (external factors)**
**Global Market Trends** In addition to the development of food products and cosmetics, including active components, proteins, functional peptides, as well as their pharmacological components, the global market is increasingly receptive to the consumption of insects [34,35]. **Policies that are consistent with and responsive to the SDGs** 1.1 Global Climate Change: A study on the promotion of edible insect production shows that it influences global climate change and food production by yielding lower greenhouse gas emissions and a reduced carbon footprint in comparison with conventional livestock production, based on the protein (grams) produced per carbon dioxide (CO_2_) emission [36]. 1.2 The fall in global production resulting from climate change which influences the entire supply chain makes food security an urgent domestic and global concern. The Thai government has established and promoted the “Thailand: cuisine to the world” scheme, aligning it with global food security efforts. **Tech-driven transparency** Innovations such as blockchain for traceability can bolster product authenticity and consumer trust [37,38].	**Confidence in edible insect products and development of production models** Insect consumption is perceived as an unfamiliar experience for many foreign consumers, particularly in European countries and the United States, leading to negative consumer imagery associated with the product. This constrains the growth of the export market [39,40]. **Confidence in safety standards** Thailand currently has a few established standards to produce edible insects and edible insect products. A singular GAP standard exists for insect production, such as dried crickets, frozen crickets, and black soldier fly (BSF) farming, announced by the National Bureau of Agricultural Commodity and Food Standards (ACFS) [41]. However, there are no established export criteria for all edible insects with economic competitive potential. Consequently, business owners and governments face challenges in advancing their industries to a level of international competitiveness [12]. **Disease-outbreak risks** High-density rearing without robust biosecurity can precipitate colony losses and reputational damage [42]. **Alternative-protein competition** Growth of plant-based “meat” analogs and cultured proteins intensifies market pressure [43].

**Table 2:** Strategic proposals to promote Thailand’s edible insect business from TOWS Matrix analysis.

**TOWS**	**Strengths (S)**	**Weaknesses (W)**
	**S1**: Various species of edible insects [23]. **S2**: Geographically and climatically appropriate [45]. **S3**: Cultural diversity and gastronomy connections [46]. **S4**: Promotion by the state [46,47].	**W1**: Traditional production—inadequate introduction of technology to control the quality and quantity of production [29]. **W2**: Uneven quantity resulted from inadequate knowledge and technology transfer [36,47] **W3**: No breeding control/genetic problems/resistance [23,47]. **W4**: Lack of promotion and development of operational patterns to increase productivity [37].
**Opportunities (O)**	**SO Strategy** **(Strength and Opportunity)**	**WO Strategy** **(Weakness and Opportunity)**
**O1**: Notable trend/sustainable and nourishing food [46]. **O2**: Policy consistent and responsive to the SDGs, climate change, carbon footprint and food security [17,47].	**SO1**: Supports the expansion of production/investment to farmers, stakeholders, and the public [23,48]. **SO2**: Promotes strategies/policies at the state and community levels, targets local sustainability, and distributes income at the community level [45,46,49]. **SO3**: Provides access to public funding, upgrading, and promoting SMEs and start-ups [50].	**WO1**: Innovate for Intelligent Breeding/Agriculture [17,37]. **WO2**: Create an Academic/Research Promoter Problem Solved [23,50]. **WO3**: Strengthen the brand or product of a company/build the image and identity of a product and consumption that is reliable and consistent with the sustainable trend [23,50].
**Threats (T)**	**ST Strategy** **(Strength and Threat)**	**WT Strategy** **(Weakness and Threat)**
**T1**: Product reliability and development of edible insect patterns. **T2**: Safety/feeding standard confidence	**ST1**: Promoting investment in R&D and innovation [50]. **ST2**: A global leader in the production and export of edible insects [23,37]. **ST3**: Promotion of local product identity in each region. **ST4**: Promotion of innovation and commercialization of edible insect products. Product Markings, establishment of a certification system by the state, promotion of international reliability [50].	**WT1**: Promoting the integration of both cooperative and large-scale agriculture to control production capacity [50]. **WT2**: Offering short courses or training in insect farming, together with the development of edible insect products through technology and innovation, can enhance human capacity for further advancements in production systems and product development. **WT3**: Supporting research to enhance knowledge and innovation [49].

**Table 3:** Management throughout the edible insect production chain.

Production	Process	Specific Operations of Relevant Units
Upstream	Rearing	Insect rearing with technology, good and adequate agricultural production standards, or GAP, food source, insect quality, production processes (location, production premises, environment, feeding equipment, water supply, food and water management, cleaning, disinfection, maintenance costs (fixed cost, variable cost, income, profit, repayment period).
Midstream	Processing	Processing and distribution, processing technology and innovation, dynamic distribution, freezing, framing, drying, powdering, insect containment, protein hydrolysis, oil extraction, and product standards.
Downstream	Marketing	Packaging, images, transportation, conservation, related legislation, online marketing, branches, production chain management, consumers, environment, tourism, insecticide integration, economy, sustainable systems, information technology, and information science.

## Abbreviations

ACFS	National Bureau of Agricultural Commodity and Food Standards
BCG	Bio-circular green economy strategy
BSF	Black soldier fly
CO_2_	Carbon dioxide
ESG	Environmental, social, and governance criteria
GAP	Good agricultural practices
IoT	Internet of things
NPK	Nitrogen, phosphorus, and potassium
RBCs	Conservation breeding programs
SDGs	Sustainable Development Goals
SEIF	Smart edible insect factory
SFA	Singapore Food Agency
SME	Small- and medium-sized enterprise
SO	Strengths and opportunities
ST	Strengths and threats
SWOT	Strengths, weaknesses, opportunities, and threats
TOWS	Threats, opportunities, weaknesses, and strengths
US FDA	The United States Food and Drug Administration
WO	Weaknesses and opportunities
WT	Weaknesses and threats
